# Assessing Diesel Tolerance of *Chromobacterium violaceum*: Insights from Growth Kinetics, Substrate Utilization, and Implications for Microbial Adaptation

**DOI:** 10.1021/acsomega.4c01698

**Published:** 2024-05-23

**Authors:** Sebastián Arenas, Nathaly Rivera, Francy Janeth Méndez Casallas, Boris Galvis

**Affiliations:** † Programa de Ingeniería ambiental y Sanitaria, Universidad de La Salle, Bogotá 110231, Colombia; ‡ Escuela de Ingeniería de los Recursos Naturales y del Ambiente—EIDENAR, Universidad del Valle, Cali 760042, Colombia

## Abstract

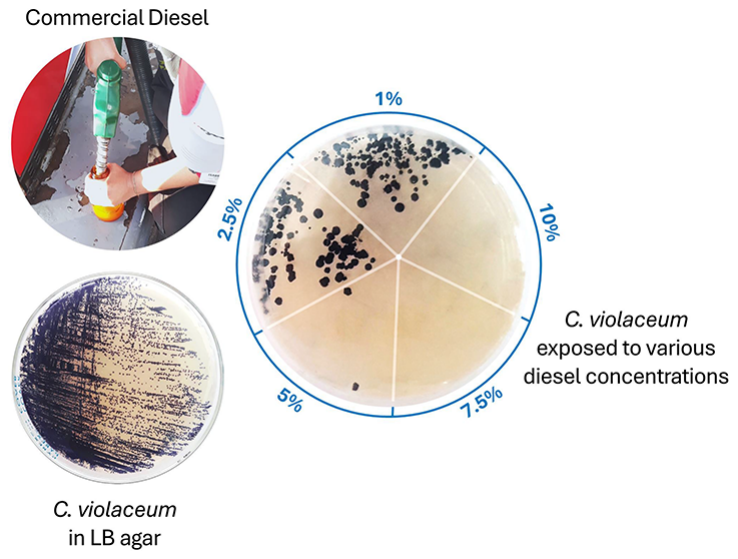

This study aimed to determine the tolerance of *Chromobacterium violaceum* ATCC 12472 to diesel. The growth of the strain was evaluated through exposure to various diesel concentrations (1, 2.5, 5, 7.5, and 10% v/v), with continuous monitoring of growth via optical density measurements until the death phase was reached. Employing a logistic model, we analyzed the growth kinetics of *C. violaceum* and compared them with five other models to comprehend substrate utilization dynamics. Our results indicate that optimal bacterial growth occurred at 2.5% (v/v) or 18,125 mg/L diesel, while both higher and lower concentrations manifested inhibitory and increasingly stressful effects. The Aiba model emerged as the most fitting representation of substrate utilization by *C. violaceum*. In addition, our findings underscore the remarkable diesel tolerance of *C. violaceum* ATCC 12472, despite the inherently stressful nature of the medium. This study contributes to the understanding of microbial responses to environmental stressors and highlights the pivotal role of the substrate concentration in influencing microbial growth. These insights have implications for bioremediation strategies and enhance our understanding of bacterial ecological resilience in the presence of hydrocarbon pollutants.

## Introduction

Hydrocarbon pollution has become increasingly common due to our growing energy demands.^1,2^ Accidental spills or leaks of crude oil and its refined products result in hydrocarbon pollution in both soil and aquatic ecosystems.^3−5^ Many of these hydrocarbons decompose slowly in the environment, and some may even be nonbiodegradable.^6,7^ Bioremediation is often a cost-efficient solution to speed up the degradation of hydrocarbon pollution.^8−10^ Microorganisms can be used to break down hydrocarbon pollutants into carbon dioxide, methane water, and biomass.^11−13^ However, microorganisms are impacted by the toxicity and low bioavailability of hydrocarbons, as well as environmental limitations, metabolic restrictions, and long recovery periods.^8,14,15^ To successfully carry out a bioremediation process, it is crucial to understand the response and tolerance of microorganisms to specific hydrocarbons, oil and fuels.^16,17^

Only a few studies have focused on determining the capacity of microorganisms to break down a mixture of pollutants containing compounds that can limit the biodegradation process, which is a common situation in the case of fuel spills.^5,15^ Diesel fuel is a complex mixture of paraffin, olefins, naphtha, and many aromatic compounds. It contains a light hydrocarbon fraction that can be toxic to many microorganisms,^18,19^ making it an interesting substrate to test the abilities of microorganisms to carry out degradation processes in the presence of harmful compounds.^20,21^

Exposure to diesel has been shown to have several adverse effects on the uniformity of microbial communities,^22^ and a decrease in species richness and phylogenetic diversity associated with the disruption of the nitrogen cycle and the significant reduction of functional species and genes involved in nitrification.^15^ Microbial kinetic parameters are a commonly used tool to assess the capacity of a species to break down diesel.^23,24^ Typically bacterial growth follows a curve with lag, exponential, and stationary phases.^25^ The main parameters of interest in the bacterial growth curve of a given microorganism are the duration of the lag phase, the asymptotic value, and the specific growth rate (μ_max_).^26^ μ_max_ and other key parameters might be used to develop secondary models that describe the effects of environmental conditions, such as pH, temperature, and pollutants, on the growth of the microorganism.^27,28^

Crude oil and processed hydrocarbons such as diesel could contain concentrations of heavy metals that can inhibit bacterial growth and affect bioremediation processes.^29,30^ Knowing if the microorganisms used for a bioremediation process can endure the presence of heavy metals and toxic petroleum compounds and at the same time degrade hydrocarbons is crucial.^5^ Various approaches have attempted to broaden the understanding of heavy metal-resistant microorganisms to use them for successful bioremediation of oil-contaminated sites.^31−35^*Chromobacterium violaceum* can withstand heavy metals and can reduce halogenated compounds to less toxic compounds.^36,37^ Previous studies have mainly focused on the adaptation of *C. violaceum* to heavy metals such as gold, copper,^38^ iron, zinc, silver, arsenic, and nickel^39^ and to a lesser extent, to platinum, aluminum, manganese, cadmium, and mercury.^40^

The sequencing of the genome of *C. violaceum* ATCC 12472 revealed that the strain presents a total of 4,751,080 base pairs and 4430 open reading frames (ORFs) of which some authors suggest could give this bacterium biotechnological applications.^36^ In addition, several of the identified ORFs were found to be involved in the stress response, suggesting that this bacterium is highly adaptable. Although the proteins that are involved in the response of *C. violaceum* to stressful environmental conditions are known, their specific roles are poorly understood.^41,42^ The presence of multidrug-resistant ORFs illustrates the contribution of membrane transport systems to the ability of *C. violaceum* to withstand environmentally unfavorable conditions. Heavy metal transporters include zntA (CV1154), which provides this bacterium with the potential for bioremediation of xenobiotics.^36^

This study modeled and analyzed the growth kinetics and substrate utilization of *C. violaceum* ATCC 12472 in batch cultures at different diesel concentrations and determined the tolerance capacity of *C. violaceum* ATCC 12472 to different concentrations of diesel.

## Materials and Methods

The study was conducted in the biotechnology laboratories of La Salle University. Diesel fuel samples were purchased at a gas station in Bogotá. The composition of Colombian diesel can be found elsewhere.^43^ Only two samples of commercial diesel were used. The first sample was used to condition the strain. A second sample of the same volume and the same gas station bought 3 weeks later was used for the biodegradation process. Possible changes in the composition of the fuel were not addressed in this study. The *C. violaceum* strain, the culture media, the Gram-staining reagents, the reagents for standardizing bacterial suspensions, and the equipment were provided by La Salle University. Proper care was taken to keep all glassware sterile to avoid contamination with chemicals and other species.

### Reactivation and Conditioning of *C. violaceum* ATCC 12472

**Figure 1:**
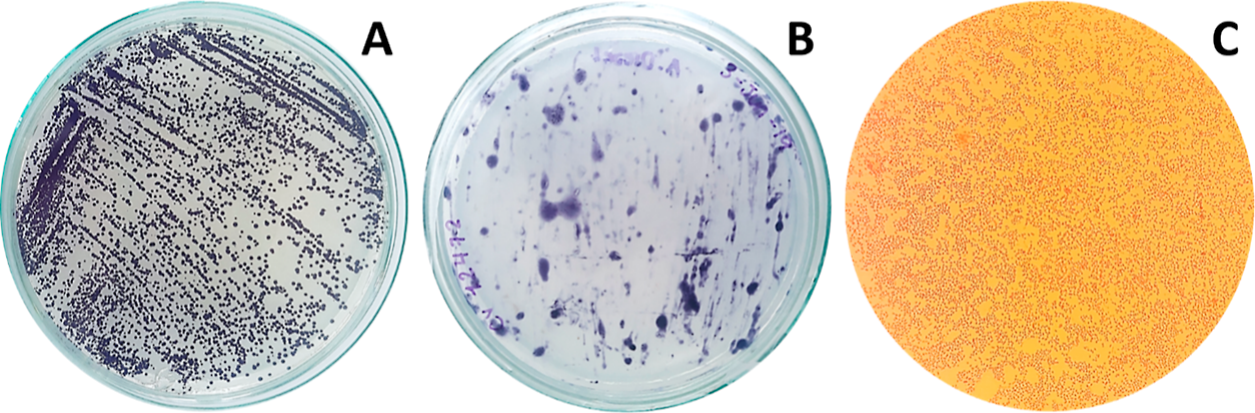
Culture of *C. violaceum* ATCC 12472 in (A) LB agar, (B) diesel agar, and (C) MSM supplemented with diesel (Gram stain observed under a microscope).

Diesel fuel samples were transported in sterile 500 mL amber Schott flasks sealed and refrigerated at 4 °C from the gas station to the laboratory. The samples were stored under low humidity conditions (approximately 52% RH) at around 17 °C and were used the next day. The pH of the fuel was approximately 5.4. The fuel dilutions were prepared using sterile 10 mL micropipettes. The fuel of the first sample was diluted in minimum salt medium (MSM) prepared as outlined below for conditioning of the bacterium. Conditioning was carried out before the biodegradation tests in MSM and diesel at a low concentration. First *C. violaceum* ATCC 12472 was reactivated in Luria–Bertani agar (LB) (Figure 1A) and incubated at 30 °C for 24 h. Later the active strain of *C. violaceum* ATCC 12472 was sown in Petri dishes with diesel-agar (MSM + agar-agar 1.5% m/v + diesel 1% v/v) (Figure 1B).

The MSM was prepared with 1 mg of CaCl_3_, 125 mg of NaHCO_3_, 70 mg of K_2_SO_4_, 70 mg of NH_4_NO_3_, 100 mg of KH_2_PO_4_, 10 mg of MgSO_4_·7H_2_O, 7 mg of MnCl_2_·H_2_O and 1.5 mg of ZnSO_4_ per liter of distilled water, following the method proposed by Narváez Flórez et al.^44^ The mixture of MSM and agar-agar was previously autoclaved to ensure sterile conditions. The culture of *C. violaceum* ATCC 12472 in diesel agar was later incubated for 48 h at 30 °C. Vials of the conditioned strain were made and stored at 4 °C in Eppendorf tubes with 1% glycerol.

### Standardization of Bacterial Suspensions and Preparation of the Inoculum

We did a McFarland turbidity curve to estimate the concentration of bacteria to be inoculated in the bioassays.^45^ The standards were done in triplicate with barium sulfate (BaSO_4_) precipitates, by adding barium chloride and sulfuric acid at different proportions as recommended by Zapata and Ramirez-Arcos.^45^ The absorbance at 600 nm wavelength of each standard was measured with a HACH DR1900 spectrophotometer (Loveland, COL, USA) and plotted against the approximate bacterial concentration according to each standard and analyzed using a linear regression model through the Statistical Software GraphPad Prism (version 8.0.1).

**Figure 2:**
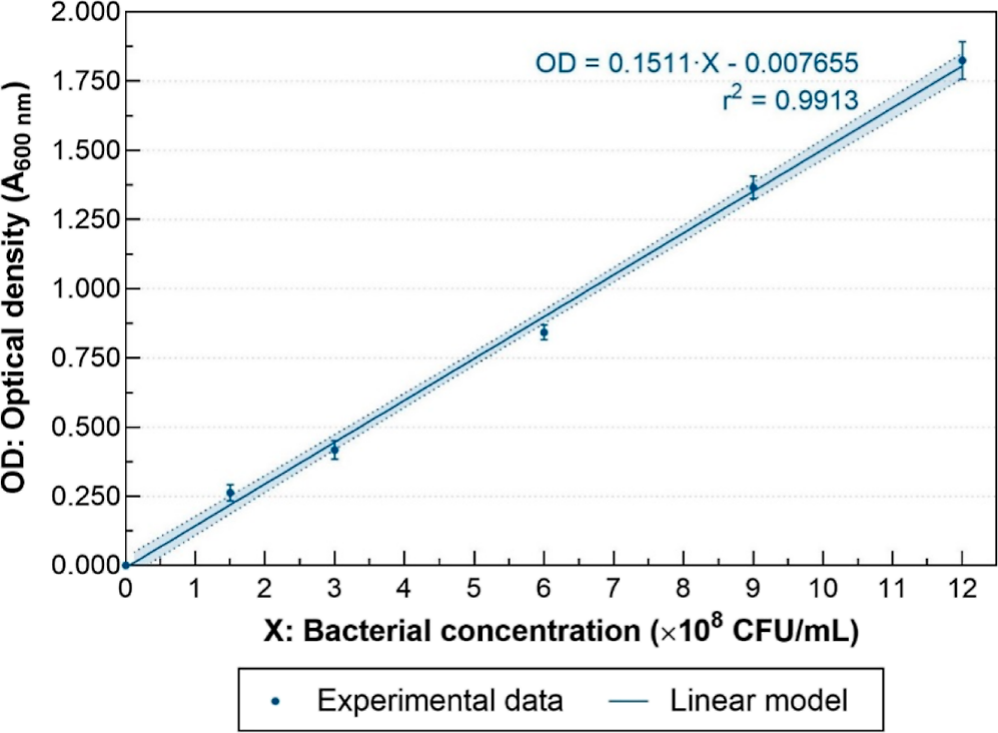
Standardization curves of the bacterial suspensions. Optical density versus bacterial concentration. The dotted lines show the 95% confidence interval.

The inoculation of *C. violaceum* ATCC 12472 was performed by placing the strain of the active culture in agar-diesel in a Schott bottle with 99 mL of sterile MSM and 1 mL of diesel. The inoculum was incubated for 24 h at 200 rpm in Thermo Fisher Scientific (Waltham, MA USA) MAXQ 4450 orbital agitator at 30 °C. After 24 h, the inoculum was diluted with sterile water until the absorbance at 600 nm corresponding to an approximate bacterial concentration of 5 × 10^8^ CFU/mL was obtained following Mendoza et al.^46^ (see Figure 2). In addition, a Gram stain was performed as a quality test to guarantee the absence of another microorganism in the inoculum (Figure 1C).

### Tolerance Tests and Growth Kinetics of *C. violaceum* ATCC 12472 in Diesel

The concentration range evaluated in the tolerance tests was between 1 and 10% (v/v) of diesel, as previously done by other authors.^44,47,48^ Considering the variability of diesel composition, the initial concentration of total petroleum hydrocarbons (TPHs) in the diesel sample was estimated to establish the tolerance of *C. violaceum* ATCC 12472 in terms of a parameter other than the percentage (volume/volume).

For the tolerance tests, 100 μL of the inoculum was added to test tubes with MSM and diesel in triplicate. The test tubes were filled to 5 mL with concentrations of 1, 2.5, 5, 7.5, and 10% (v/v) to allow the oxygenation of the sample in a 1:1 ratio. The cultures were incubated at 30 °C in the orbital agitator at 200 rpm^44^ until their death phase. We measured the absorbance at 600 nm of each tube daily as done by Ahmed et al.,^49^ using the HACH DR1900 spectrophotometer. Before the measurement, each tube was agitated at 50 rpm for 30 s, guaranteeing that no diesel emulsions were formed. These tests allowed us to evaluate the growth kinetics of *C. violaceum* ATCC 12472 in different diesel concentrations. We also determined the growth phases of each treatment and analyzed the significant differences between them.

### Modeling the Growth Kinetics of *C. violaceum* ATCC 12472 in Diesel

The growth kinetics of *C. violaceum* ATCC 12472 were modeled by applying the logistic model^50^ (eq 1), using the statistical software GraphPad Prism (version 8.0.1). The equation relates the bacterial concentration (*X*), maximum (max), and initial (0), the specific growth rate (μ), and the incubation time (*t*).
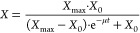
1The model allows us to estimate μ, generation time (*t*_g_), and maximum yield (*M*). LB broth was used as a reference medium for a positive control because of its favorable nutrient conditions. To determine the kinetics in LB broth, a reactivated colony of *C. violaceum* ATCC 12472 was taken from a vial without prior contact with diesel in 5 mL of saline solution. The liquid culture was diluted to obtain the required absorbance at 600 nm to obtain a bacterial concentration of approximately 5 × 10^8^ CFU/mL, and 100 μL were inoculated in three test tubes with 5 mL of sterile LB broth each. The LB broth cultures were incubated for 24 h under the same conditions as the diesel cultures, and spectrophotometric measurements at 600 nm were made every hour. Subsequently, its kinetics were modeled by the logistic model, and their kinetic parameters were obtained. The absorbance of the salts was subtracted by using daily averages from the abiotic control.

Finally, to evaluate whether the treatments of *C. violaceum* ATCC 12472 and the positive control, presented significant differences between the growth parameters evaluated, we did an analysis of variance using the IBM SPSS Statistics software v25 (see Supporting Information).

### Modeling of Diesel Utilization Kinetics by *C. violaceum* ATCC 12472

We also modeled the kinetics of substrate utilization to evaluate the effect of the diesel concentration on the growth rate of *C. violaceum* ATCC 12472. TPH analyses were performed at an independent lab. We used the Monod, Teissier, Haldane, Aiba, and Luong models to evaluate substrate utilization. Mathematical expressions are presented in Table 1. Additionally, these models were used to estimate the optimal diesel concentration range for the growth of *C. violaceum* ATCC 12472.

**Table 1:** Kinetic Models of Substrate Utilization^a^

model	equation		reference
Monod	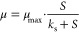 2	2	(51)
Teissier	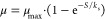 3	3	(52)
Haldane	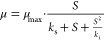 4	4	(53)
Have	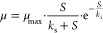 5	5	(54)
Luong	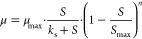 6	6	(55)

a Symbols correspond to specific growth rate (μ), specific maximum growth rate (μ_max_), substrate concentration (*S*), maximum substrate concentration (*S*_max_), saturation constant (*k*_s_), inhibition constant (*k*_i_), and Luong model parameter (*n*).

### Validation of Models

We used different metrics to assess how well the models reproduce the experimental data. We estimated the coefficient of determination (*R*^2^), and the adjusted coefficient of determination (*R*_adj_^2^) as suggested by Dahalan and Ali Hassan.^56^ Additionally, we estimated the sum of squares (SS), the root mean square error (RMSE), and the Akaike information criterion (AIC).^57^ Values of *R*^2^ and *R*_adj_^2^ closer to 1 and smaller SS, RMSE, and AIC indicate better model performance.

## Results and Discussion

### Standardization of Bacterial Suspensions

Standardization curves of bacterial suspensions (Figure 2), obtained from the prepared McFarland standards, show good agreement (*R*^2^ of 0.99) between optical density and the bacterial concentration. We learned that an optical density of 0.748 ± 0.024 units at 600 nm is equivalent to 50 × 10^7^ CFU/mL. We used this value for the spectrophotometric adjustment of the inoculum. The McFarland scale to quantify the bacterial concentration of *C. violaceum* in liquid suspensions is widely used because it is fast and simple.^58−61^

### Growth Kinetics of *C. violaceum* ATCC 12472 in Batch Cultures at Different Diesel Concentrations

**Figure 3:**
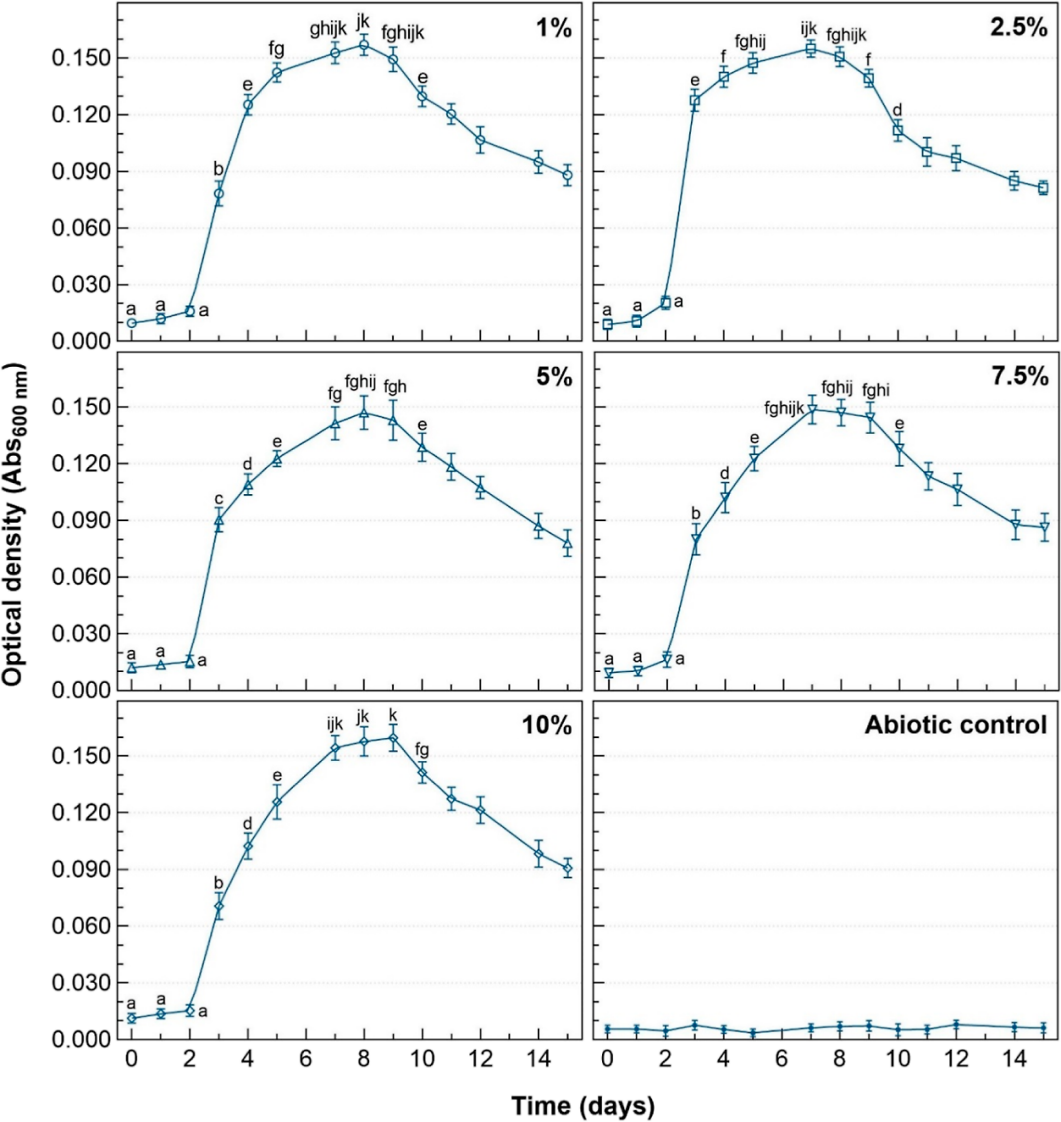
Growth of *C. violaceum* ATCC 12472 in batch cultures at 30 °C and 200 rpm for different diesel concentrations. Different letters correspond to significant differences between measurements (*p* < 0.05).

Growth of *C. violaceum* ATCC 12472 in batch cultures at 30 °C and 200 rpm with diesel concentrations between 1 and 10% (v/v) (7250 and 72,500 mg_TPHs_/L respectively) showed similar behavior between them (see Figure 3). All cultures exhibited the normal phases. All cultures displayed lag and acceleration phases lasting approximately 2 days. On the third day, all cultures reached the exponential phase. Duncan’s test (*p* < 0.05) (see the Supporting Information) indicated that bacteria growing in 2.5 and 5% (v/v) diesel had significantly higher growth on the third day. The duration of the exponential phase was around 2 days for all cultures, except for 2.5 and 10% (v/v) in which it took 1 and 3 days, respectively. Both high concentrations (>2.5% v/v) and low concentrations (<2.5% v/v) of diesel decrease the exponential growth rate of *C. violaceum* ATCC 12472, resulting in the prolongation of the exponential phase. As for the deceleration phase, its duration was longer in cultures with high concentrations of diesel. For cultures with 1 and 2.5% (v/v) diesel, the deceleration phase lasted almost 1 day, while for cultures with 5% and higher, it took approximately 2 days. A gradual decrease in the optical density of all cultures was observed from the ninth day (eighth day for the culture with 2.5% v/v of diesel) until the last day monitored. This decline was associated with the completion of the stationary phase and the initiation of the death phase. The stationary phase was shorter in cultures with high concentrations of diesel, contrary to what was observed for previous phases. The duration of the stationary phase was close to 4 days for cultures with 1 and 2.5 (v/v) diesel, 3 days for cultures with 5 and 7.5% (v/v), and only 2 days for 10% (v/v). The maximum bacterial densities observed in cultures with 1, 2.5, and 10% (v/v) diesel were slightly higher than those of cultures with 5 and 7.5% (v/v) of diesel. Variations observed in the duration of the growth phases, the exponential growth rates, and the maximum bacterial densities between the cultures of *C. violaceum* ATCC 12472 at different concentrations of diesel allow us to better understand the growth response of this microorganism to this fuel.

Lag phases of *C. violaceum* ATCC 12472 at different diesel concentrations all lasted 2 days. This behavior is dissimilar to that of other bacteria such as *Pseudomonas putida* TPHK-1, *Stenotrophomonas maltophilia* TPHK-2, and *Acinetobacter* sp. TPHK-3 and *Pseudomonas aeruginosa* TPHK-4. For these bacteria, cultured between 1 and 5% v/v (8000–40,000 mg/L) of diesel, concentrations greater than 3% v/v (24,000 mg/L) prolonged the lag phase.^5^ In general, the duration of the lag phase depends on several factors such as the size of the inoculum, the physiological history of the cells, and the specific physicochemical conditions of both the original and the new culture medium.^62^ Due to the multiple changes and adaptations that occur throughout it, lag phase duration has been directly related to cellular stress, being used as a direct measure of growth inhibition induced by the environmental conditions of the culture medium.^10^

Diesel is a hostile environment for microorganisms, significantly affecting their normal growth.^18^ In a diesel medium, sufficient time is needed for bacteria to metabolically adjust their cellular machinery for the synthesis of hydrocarbon-degrading enzymes,^15^ and maximize the import and processing of the primary resources they will use in the next phase.^63^ Therefore, variations in the duration of the lag phase with an increase in diesel concentrations could be expected. According to Hamill et al.,^64^ often cellular stress can prolong the lag phase, and also cause a decrease in the exponential growth rate for a given microbial population; however, it depends on the type and intensity of stress and the ability of the species to respond and adapt to such a condition. They also mention that both effects do not necessarily occur and report cases of a decrease in growth rate without lengthening the lag phase or a prolonged lag phase with an unchanged growth rate. In the present study, we observed that the high concentrations of diesel did not cause prolongation of the lag phase of *C. violaceum* ATCC 12472 but a decrease in its growth rate.

The effect of stress could be more related to the biophysical and/or physicochemical constraints that occur during the exponential phase than to the initial adaptation processes. When growing exponentially, the cells configure their machinery to maximize reproduction.^65,66^ Despite this, bacteria rarely reach their theoretical maximum growth rate due to oxidative stress, nutrient shortages, inhibitors of cell division, and environmental stress, among other factors; hence the rate of growth in this phase could be an indicator of stress in the system.^64^ In this sense, the concentration of diesel influences the exponential growth of *C. violaceum* ATCC 12472, either by the inhibitory effect of high concentrations or by limiting the amount of carbon with low concentrations.

Duration of the exponential phase of *C. violaceum* ATCC 12472 cultures in diesel are similar to those observed in *P. putida* TPHK-1 and *P. aeruginosa* TPHK-4 by Ramadass et al.^5^ They reported lower growth rates, as well as longer exponential phases for cultures with concentrations less than 2% v/v (16,000 mg/L) and greater than 3% v/v (24,000 mg/L) of diesel.

Halmi et al.^28^ found higher growth rates of *Acinetobacter* sp. DRY12 in diesel in cultures with 1 and 2% v/v. The duration of the exponential phase was shorter in cultures with 0.5 and 6% v/v diesel than those with lower and higher concentrations (0.1 and 8 to 10% v/v respectively). More recently, Jimoh and Lin^47^ evidenced that the exponential growth of *Paenibacillus* sp. D9 occurred from day 1 to 10 in cultures with 1 and 2% (v/v) diesel, unlike cultures with 5 and 10% (v/v) diesel, which lasted until day 18.

Concerning the duration of the exponential phase, it is also important to consider that, in these closed systems, high growth rates cause a faster decrease in nutrients and an increase in the accumulation of toxic byproducts;^65^ which lead to the slowing of growth and therefore to the earliest completion of said phase. The accumulation of metabolic products has been found to limit the growth of some populations even when the substrate is still available; specifically during hydrocarbon biodegradation, the accumulation of fatty acids can block the metabolic activity of microorganisms.^6^ Xu et al.^15^ reported that in the biodegradation of petroleum hydrocarbons by bacteria, some intermediate metabolites with relatively high solubility are produced, which may have cytotoxicity greater than that of the original molecules, causing damage to the cells.

The duration of the deceleration phase is also shortened under optimal diesel concentrations. This is consistent with the observed growth profile of the *Burkholderia* sp. DRY27, where the deceleration phase was prolonged in cultures with diesel concentrations below 1% v/v (8500 mg/L) and above 4% v/v (34,000 mg/L). In addition, in high concentrations, the prolongation was greater with larger concentrations.^30^ Given that the degradation mechanism of diesel and biodiesel is related to the production of biosurfactants by both *C. violaceum*(67) and *Burkholderia* sp.,^68^ the degradation kinetics might be comparable.

The deceleration phase can be also prolonged in complex media because the stress induced by the depletion of nutrients or the accumulation of waste causes a restructuring of the cell to increase the prospects of cell survival in a hostile environment making it more time-consuming.^65^ From this restructuring given in the deceleration phase, the microbial population can employ alternative sources during the stationary phase. The duration of the stationary phase depends on the ability of the microorganism to use these alternative sources.

We found that high diesel concentrations (>2.5% v/v) shortened the duration of the stationary phase of *C. violaceum* ATCC 12472. However, the effect of diesel concentration was different for biomass production, as concentrations between 5 and 7.5% (v/v) showed a reduction in bacterial density but not a reduction for a concentration of 10% (v/v). We believe that the metabolic demands represented by the concentration of 10% (v/v) diesel induced the bacterial population to activate mechanisms different from those of lower concentrations during the lag phase; allowing them to maximize the production of biomass in the early stages but limiting the mechanisms to be used in the stationary phase and making it shorter.

It should be noted that in the lag phase, the internal machinery of the cells adapts to the new environmental conditions, so new enzymes are synthesized, and the synthesis of others is repressed depending on the composition of the new culture medium.^65^ The expression of genes during the lag phase allows adjusting the bacterial metabolism to ensure maximum biomass gain after finishing this phase, focusing on the production of bottleneck enzymes for carbon utilization.^63^

Three homogeneous subsets can be identified in the six treatments using an analysis of variance: the first corresponding to the control group (abiotic control), the second consisting of cultures with 5 and 7.5% (v/v) diesel, and the third by cultures with 1, 2.5, and 10% (v/v) diesel (see the Supporting Information). These groups are related to the differences in the maximum bacterial densities already mentioned.

### Modeling of the Growth of *C. violaceum* ATCC 12472 in Diesel

**Figure 4:**
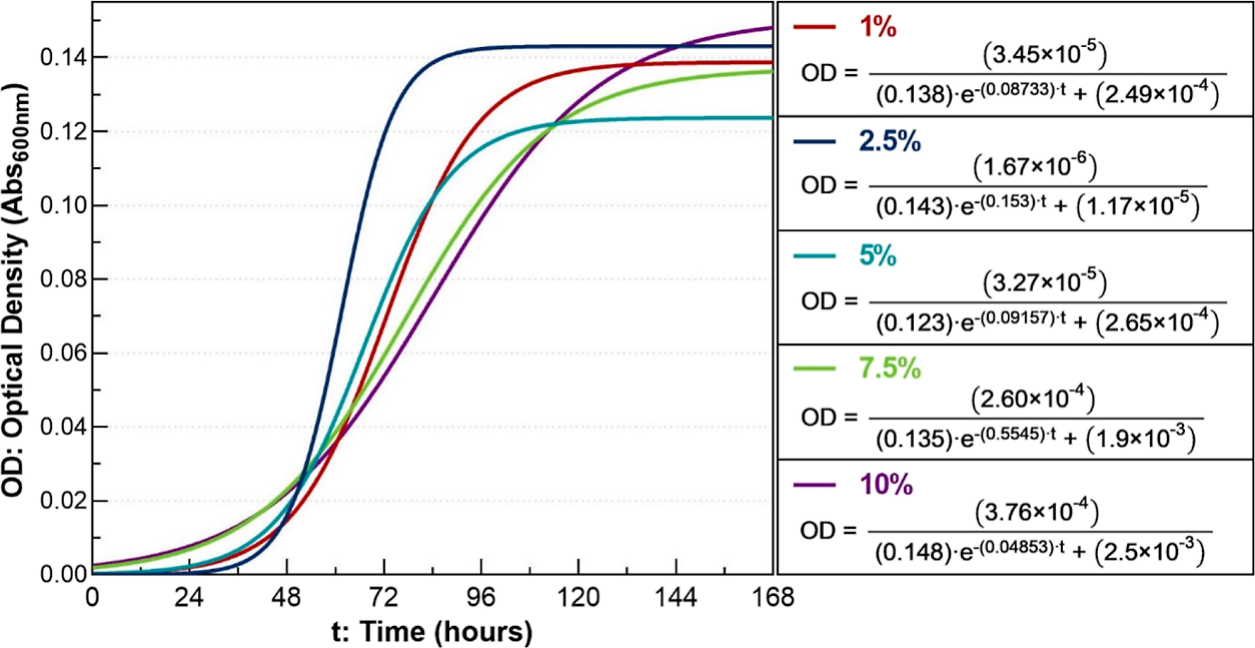
Growth kinetics of *C. violaceum* ATCC 12472 in batch cultures at 30 °C and 200 rpm with diesel at different concentrations using the logistic model: 1 (*R*_adj_^2^ = 0.9917), 2.5% (*R*_adj_^2^ = 0.9922), 5% (*R*_adj_^2^ = 0.9669), 7.5% (*R*_adj_^2^ = 0.9680) and 10% (*R*_adj_^2^ = 0.9832).

We modeled the growth kinetics of *C. violaceum* ATCC 12472 at different diesel concentrations using the logistic model (Figure 4). The model gives insight into the effect of the diesel concentration on the growth of *C. violaceum* ATCC 12472. There are clear differences between the slopes of the curves and the maximum values of bacterial density between the treatments. The treatment with 2.5% (v/v) diesel has the highest growth rate.

**Figure 5:**
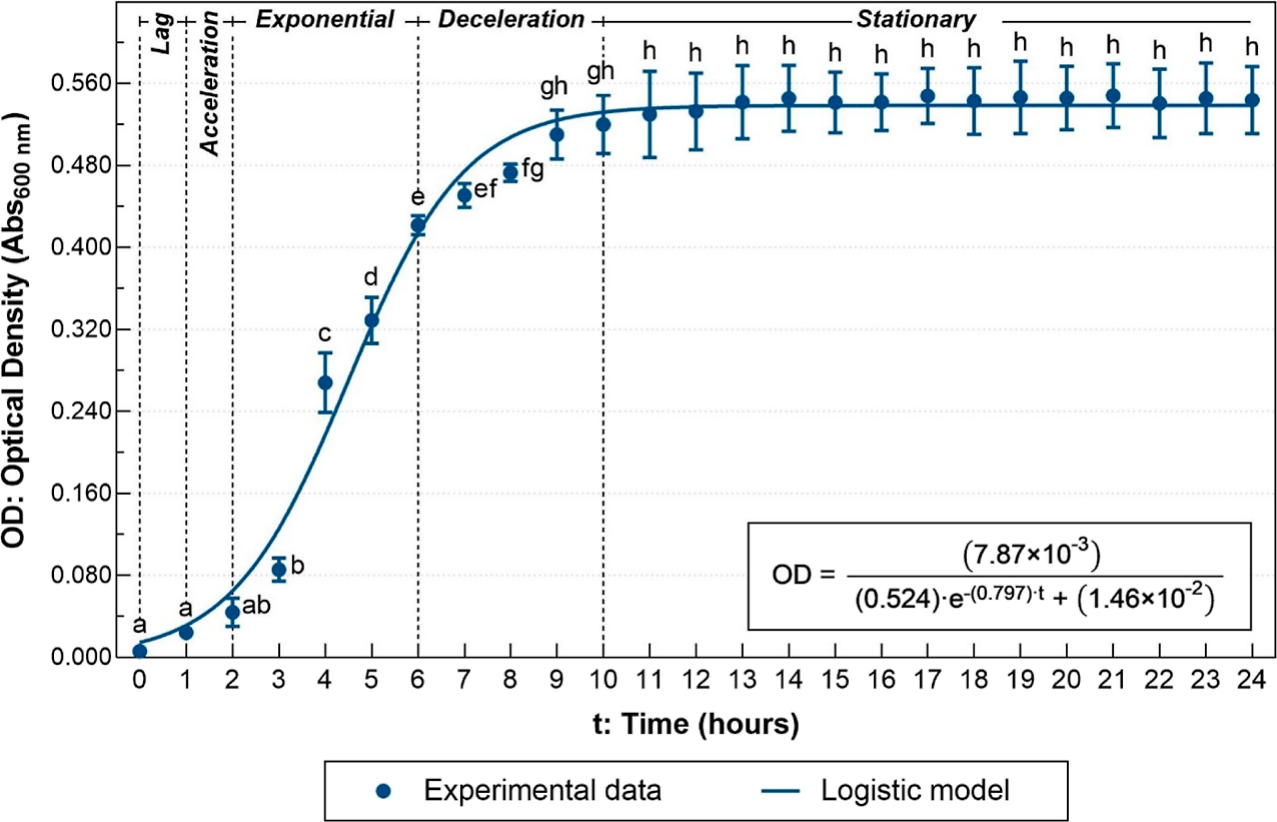
Growth kinetics of *C. violaceum* ATCC 12472 in LB broth at 30 °C and 200 rpm for 24 h. The continuous line corresponds to the logistic model (*R*_adj_^2^ = 0.9758). Different letters correspond to significant differences between measurements (*p* < 0.05).

The positive control culture of *C. violaceum* ATCC 12472 in LB broth showed the first five phases of growth during the 24 h of their incubation (see Figure 5). The culture took approximately 1 h to adapt to the new environment and 2 h to start its exponential growth. The deceleration phase occurred between hours 6 and 10, and after came the stationary phase that remained until the 24th hour. We observed reductions of 87 ± 8 and 74 ± 3% in the speed of growth and maximum yield in cultures with diesel when compared to LB broth, respectively.

The coefficients μ, *X*_0_, and *X*_max_ given by the logistic model allow us to analyze the effects of the type and quantity of the carbon source on bacterial growth through.^69^ They are related to the kinetic parameters of growth: specific growth rate (μ), generation time (*t*_g_), and maximum yield (*M*). Table 2 shows the estimated values of these growth parameters in part *C. violaceum* ATCC 12472 cultures in both diesel media and LB broth.

**Table 2:** Kinetic Parameters of the Growth of *C. violaceum* ATCC 12472 in Batch Cultures with Diesel at Different Concentrations and LB Broth at 30 °C and 200 rpm^a^

middle	μ (h^–1^)	*t*_g_ (h)	*M* (Abs_600nm_)
MSM + diesel (1% v/v)	0.09 ± 0.02^ab^	7.97 ± 1.40^b^	0.139 ± 0.006^bc^
MSM + diesel (2.5% v/v)	0.15 ± 0.02^b^	4.55 ± 0.66^b^	0.143 ± 0.003^bc^
MSM + diesel (5% v/v)	0.10 ± 0.03^ab^	7.77 ± 2.67^b^	0.124 ± 0.006^a^
MSM + diesel (7.5% v/v)	0.06 ± 0.01^a^	12.59 ± 3.20^a^	0.135 ± 0.009^b^
MSM + diesel (10% v/v)	0.05 ± 0.01^a^	14.36 ± 2.10^a^	0.148 ± 0.004^c^
LB broth	0.80 ± 0.01^c^	0.87 ± 0.09^c^	0.523 ± 0.002^d^

a Different letters correspond to significant differences between treatments (*p* < 0.05).

The specific growth rate of *C. violaceum* ATCC 12472 was higher in cultures with concentrations between 1 and 5% (v/v) diesel and showed a maximum value of 2.5% (v/v). As expected, the minimum value of *t*_g_ was also obtained at the same concentration, and higher or lower concentrations increased its value. Maximum yield increased for a diesel concentration of 2.5% (v/v). It decreased drastically with 5% (v/v) and increased again with higher concentrations (>5% v/v).

An analysis of variance showed significant differences in the kinetic parameters (Supporting Information). All growth parameters of *C. violaceum* ATCC 12472 in LB broth were significantly different from those of cultures with diesel in all cases. The specific growth rate culture with 2.5% (v/v) is significantly different from all other treatments and from the LB broth. Treatments with 1, 2.5, and 5% (v/v) show essentially the same *t*_g_ but have a significantly different *t*_g_ to treatments with 7.5 and 10% (v/v) and LB broth. Finally, cultures with 5, 7.5, and 10% (v/v) diesel show significantly different maximum yields. 1 and 2.5% (v/v) diesel show similar maximum yields to 7.5 and 10% (v/v) diesel.

We observed an initial increase in the specific growth rate of *C. violaceum* ATCC 12472 with a rising diesel concentration until it reached a critical peak value. This modeling result aligns with observations that concentrations exceeding 2.5% (v/v) or 18,125 mg/L of diesel inhibit the strain’s growth.

Our findings confirm that diesel-contaminated mediums create a stressful environment for *C. violaceum* growth. The availability of diesel may explain the significant reduction in the maximum growth rate and yield of diesel cultures compared with LB broth cultures. The limited aqueous solubility of carbon sources, particularly hydrocarbons like diesel, can impede bacterial growth due to mass transfer restrictions.^69,70^ Additionally, the bacterial population increase may be constrained when the concentration of the carbon source falls below the minimum limit. In our experiment, diesel served as the sole carbon source, and both low and high concentrations of this substrate hindered the strain’s development. The inhibition of microbial growth at high concentrations could be attributed to the lipophilic nature of hydrocarbons.^44^

High concentrations of diesel in bacterial cultures exert selective pressure and toxicity, disrupting cell membranes and leading to alterations in nutrient transfer, macromolecule release, and ultimately cell death.^30,47,48,56^ This solvent effect of diesel is linked to a decrease in the proton motive force within cells.^18^ The concentration of diesel plays a pivotal role in biodegradation, with microorganisms thriving when their growth rate is below the dissolution rate of hydrocarbons.^47^ Consequently, high diesel concentrations may hinder the specific growth rate, while low concentrations could limit growth due to insufficient contact between diesel and microorganisms.^48,56^

Concentrations exceeding 2% (v/v) of diesel typically result in low growth rates,^47^ potentially reaching zero, as observed in *Pseudomonas* sp. DRYJ3 at 4% (v/v) diesel, where bacterial growth ceased entirely.^56^ The maximum growth rate of *C. violaceum* ATCC 12475 in diesel is comparable to that of *Pseudomonas* sp. DRYJ3 (0.16 h^–1^) and *Rhodococcus pyridinivorans* F5 (0.20 h^–1^), and higher than those of *Pseudomonas citronellolis* (0.18 day^–1^), *Micrococcus luteus* (0.11 day^–1^), *Serratia marcescens* (0.15 day^–1^), and *Pseudomonas* sp. (0.08 day^–1^).^56,71−73^

The type of carbon source plays a crucial role in activating metabolic pathways and inducing the production of specific enzymes in cells.^69^ Hydrocarbons, such as those found in diesel, are known to trigger a stress response leading to changes at the membrane, enzyme, and protein levels in cells.^44^ In microbial cultures with diesel, it has been observed that this substrate stimulates the production of various enzymes, including lipases, catalases, hydrolases, laccases, manganese peroxidases (MnP), and dehydrogenases, along with the activation of pathways like the β-oxidation of fatty acids^74−76^

Chandran and Das^77^ discovered an increase in the activity of enzymes like cytochrome P450 monooxygenase, aminopyrine *N*-demethylase, and NADPH cytochrome-C reductase in microorganisms growing in diesel. Conversely, Sowani et al.^78^ observed that cells of *Gordonia amicalis* HS-11 grown with diesel exhibited higher surface hydrophobicity compared to growth in LB broth.

*C. violaceum* ATCC 12472 exhibits a higher total protein production under stressful conditions compared with reference conditions. However, the diversity of proteins is reduced in stressful environments. This suggests that the strain increases overall protein production to adapt to challenging conditions while minimizing the expression of unnecessary proteins.^79^ Nevertheless, the metabolic response is influenced by the type of carbon source and its concentration in the culture medium. Bacteria can adjust their metabolic and enzymatic activities in response to environmental conditions.^80^

In the case of diesel, high concentrations (10% v/v) can either lead to bacterial death or trigger various biological and metabolic activities. Some bacteria tend to produce more biosurfactants in response to the increasing diesel in the culture medium. *Paenibacillus* sp. D9, for instance, showed the highest production of lipopeptide biosurfactants at 10% (v/v) diesel, despite the observed toxicity of this concentration to the bacterium.^47^

Regarding *C. violaceum* ATCC 12472, its protein expression response varies depending on the specific environmental pressures. The hypothesis is that beyond 5% (v/v), the selective pressure of the medium compels the bacterium to employ alternative mechanisms for growth, achieving similar biomass levels as cultures with 1 and 2.5% (v/v) but with lower growth rates.^79^ This variability may explain the observed differences in the maximum yields of our diesel cultures.

### Morphology Changes in *C. violaceum* ATCC 12472 Cultivated in Diesel

Diesel not only impacted the growth kinetics of *C. violaceum* ATCC 12472 cultures but also induced changes in the macroscopic colony morphology and the intensity of violacein pigmentation. As seen in Figure 1A,B, diesel cultures exhibited larger colonies in smaller numbers compared to LB agar with slightly lighter coloration. Microorganisms can display different colony forms based on the type of culture medium used.^81^

For many microorganisms, growth in hydrocarbons can lead to morphological variations compared to growth in carbohydrates. For instance, microscopic differences in bacterial cultures have been noted between reference and diesel media, as seen in species like *G. amicalis* HS-11^78^ and *Acinetobacter haemolyticus* Zn01.^82^ In the case of *G. amicalis* HS-11, Sowani et al.^78^ observed thinner, elongated, and clustered cells with smooth surfaces in a diesel-supplemented medium, contrasting with the typical rod-shaped cells with rough surfaces in LB broth.

Castro et al.^79^ noted that *C. violaceum* ATCC 12472 cultures under reference conditions exhibit a dark violet metallic luster, while nutrient-poor and stress-inducing conditions may cause a loss of characteristic pigmentations. The alteration in pigmentation in these cultures primarily stems from a decreased expression of proteins involved in violacein biosynthesis.

### Modeling Diesel Use by *C. violaceum* ATCC 12472

**Figure 6:**
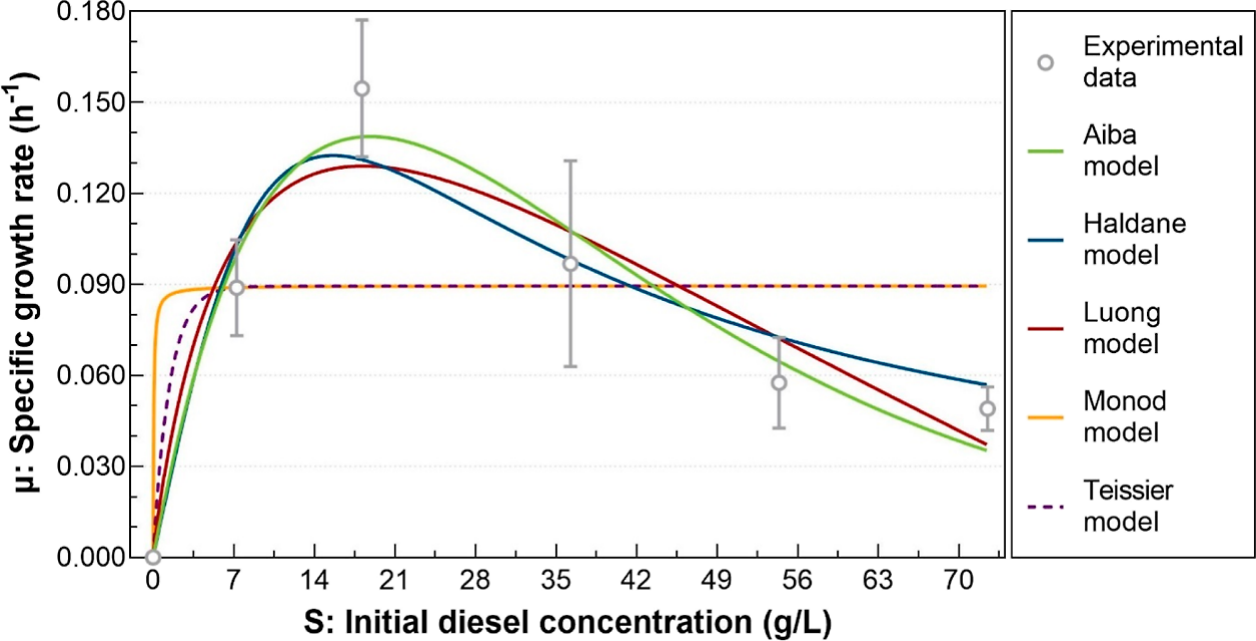
Kinetics of diesel use by *C. violaceum* ATCC 12472 using five different models.

We used five models to analyze the use of diesel by *C. violaceum* ATCC 12472 in batch cultures. Table 3 shows the kinetic constants obtained for each of the models. The Aiba, Haldane, and Luong models provided a better fit than the Monod and Teissier models, as noted in the error metrics in Table 4 and depicted in Figure 6. The Aiba model shows the highest values of *R*^2^ and *R*_adj_^2^ and the lowest values of SS, RMSE, and AIC, followed by the Haldane and Luong models.

**Table 3:** Kinetic Constants Obtained from the Use of Diesel by *C. violaceum* ATCC 12472 in Batch Cultures^a^

model	μ_max_ (h^–1^)	*k*_s_ (% v/v)	*k*_i_ (% v/v)	*S*_max_ (% v/v)	*n*
Aiba	1.477	72.04	23.79		
Haldane	1.972	108.50	2.25		
Luong	0.973	19.70		93.19	0.1977
Monod	0.090	0.05			
Teissier	0.089	1.41			

a Symbols correspond to specific maximum growth rate (μ_max_), saturation constant (*k*_s_), inhibition constant (*k*_i_), maximum substrate concentration (*S*_max_), and Luong model parameter (*n*).

**Table 4:** Error Metrics of the Kinetic Models of Diesel Use by *C. violaceum* ATCC 12472 in Batch Cultures^a^

model	*R*^2^	*R*_adj_^2^	SS	RMSE	AIC
Aiba	0.8546	0.8352	0.00658	0.01967	–131.4
Haldane	0.8343	0.8122	0.00749	0.02099	–129.0
Luong	0.8136	0.7737	0.00843	0.02226	–123.0
Monod	0.4399	0.4049	0.02532	0.03860	–110.5
Teissier	0.4415	0.3218	0.02525	0.03854	–103.2

a We estimated the coefficient of determination (*R*^2^), adjusted coefficient of determination (*R*_adj_^2^), sum of squares (SS), root mean square error (RMSE), and Akaike information criterion (AIC). Values of *R*^2^ and *R*_adj_^2^ closer to 1 and smaller SS, RMSE, and AIC indicate a better model performance.

The Haldane model is commonly used for modeling substrate inhibition in bacterial growth or biodegradation rates due to its general adjustability.^56^ Numerous studies on diesel biodegradation have reported that the Haldane model best describes the growth kinetics of degrading microorganisms in this medium.^30,48,71,73^ Even though, the Aiba and Luong models have proven effective in contaminant biodegradation studies, as noted by Karamba et al.^83^ Khoshdel and Vaziri^84^ compared the Haldane and Aiba models in the context of a microbial community’s growth kinetics during the biodegradation of aliphatic hydrocarbons and polycyclic aromatic hydrocarbons and Aiba’s model emerged as the superior predictor of the bioremediation process. Diesel biodegradation by *Pseudomonas* sp. DRYJ3 was analyzed by Dahalan and Ali Hassan^56^ using seven kinetic models of substrate utilization: Monod, Teissier, Haldane, Aiba, Luong, Yano–Koga, and Han–Levenspiel. Their findings indicated that the Luong model exhibited the best fit, followed by the Aiba model. They highlighted limitations in the Haldane model, particularly when the specific growth rate reaches zero at very high substrate concentrations due to the total inhibition of bacterial growth.

The Monod model falls short when substrates, such as diesel, exhibit inhibitory effects on bacterial growth. Both the Monod and Teissier models cannot represent microorganisms’ responses to environmental variations and the generation of different products and byproducts inherent in bacterial metabolism.^56^

Bacterial growth inhibition can occur due to various factors, including the presence of inhibitory compounds in the culture medium, such as phenols, polycyclic aromatic hydrocarbons, or heavy metals. These compounds can disrupt microbial metabolism. Additionally, the inhibitory effect can sometimes stem from the same substrate or its products. The decrease in the microbial growth rate in hydrocarbons depends on both the concentration of these compounds and their inherent toxic properties, where higher toxicity is associated with lower solubility.

Diesel is composed of low molecular weight compounds, known for their higher toxicity compared to long-chain hydrocarbons due to increased solubility and bioavailability.^85^ The cellular damage caused by diesel is primarily attributed to its low molecular weight hydrocarbons, unsaturated compounds, aromatic compounds, and acids.^18^ High concentrations of diesel not only inhibit growth by interfering with cell membrane permeability but also disrupt cellular communication mechanisms, and in the case of *C. violaceum*, this leads to a decrease in violacein production (Ramadass et al., 2017).

Making distinctions between inhibition models is challenging, as highlighted by Kim et al.^86^ The debate among authors regarding the best model to represent a substrate’s inhibitory effect on bacterial growth is ongoing. However, it is essential to note that the suitability of a specific model depends on the microorganism being studied and the prevailing environmental conditions.

### Maximum Tolerance Concentration of *C. violaceum* ATCC 12472 to Diesel

*C. violaceum* ATCC 12472 exhibited growth across all of the evaluated concentrations. We established experimentally that its maximum tolerance to diesel is 10% (v/v) or 72,500 mg/L. However, the study suggests the potential for evaluating growth kinetics at higher concentrations. Referring to the Luong model, known for predicting a microorganism’s maximum tolerance concentration to a substrate, it is estimated to be around 12.85% (v/v) or 93,190 mg/L.

Compared with other bacteria, *C. violaceum* ATCC 12472 demonstrates a maximum tolerance concentration higher than that of *Acinetobacter* sp. DRY12, *Acinetobacter* sp. TPHK-3, and *Burkholderia* sp. DRY27, *P. aeruginosa* TPHK-4, *P. putida* TPHK-1, and *S. maltophilia* TPHK-2 (see Table 5). However, it is lower than the reported tolerance of *P. citronellolis* KHA and an unidentified Gram-negative coccus isolated from contaminated soil in Bangladesh, which tolerated up to 25% (v/v) of diesel.^49^

**Table 5:** Comparison of the Reported Maximum Concentration Tolerance and Optimal Growth Concentration of *C. violaceum* ATCC 12472 in Diesel with Other Diesel Degrading Bacteria

bacteria	range evaluated (% v/v)	MCT		OGC		reference
		(% v/v)	(g/L)	(% v/v)	(g/L)	
*C. violaceum* ATCC 12472	1.0–10.0	10.0	72.5	2.0–3.3	14.5–23.9	present study
Coco Gram-negative (unidentified)	5.0–25.0	25.0		20.0		(49)
*P. citronellolis* KHA	1.0–15.0	15.0	126.0	2.0–3.0	16.8–25.2	(73)
*Paenibacillus* sp. D9	1.0–10.0	10.0		1.5–2.0		(47)
*R. pyridinivorans* F5	1.0–10.0	9.0		1.5–2.5		(71)
*Acinetobacter* sp. DRY12	0.5–10.0	8.0	68.0	3.0–5.0	25.5–42.5	(48)
*Burkholderia* sp. DRY27	0.5–7.0	7.0	59.5	2.0–3.0	17.0–25.5	(30)
*Acinetobacter baumannii*	1.0–5.0	5.0		4.0		(87)
*Staphylococcus aureus* DRY11	1.0–5.0	5.0		4.0		(88)
*P. putida* TPHK-1	1.0–5.0	5.0	40.0	3.0	24.0	(5)
*P. aeruginosa* TPHK-4	1.0–5.0	5.0	40.0	2.0	16.0	
*S. maltophilia* TPHK-2	1.0–5.0	5.0	40.0	1.0	8.0	
*Acinetobacter* sp. TPHK-3	1.0–5.0	5.0	40.0	1.0	8.0	
*Pseudomonas* sp. DRYJ3	0.5–4.0	4.0		3.5		(89)
	0.2–4.0	3.4		0.4–0.6		(56)

Our study also revealed that diesel concentrations between 2.0 and 3.3% (v/v), equivalent to 14,500 and 23,900 mg/L, favor the growth of *C. violaceum* ATCC 12472, as indicated by the Aiba model. The optimal diesel concentration range for the growth of *C. violaceum* ATCC 12472 aligns with that of several reported species, including *Burkholderia* sp. DRY27, *P. aeruginosa* TPHK-4, *P. citronellolis* KHA, and *P. putida* TPHK-1. It is larger than that of *Acinetobacter* sp. TPHK-3 and *S. maltophilia* TPHK-2, and smaller than that of *Acinetobacter* sp. DRY12 (Table 5).

Typically, the diesel tolerance of hydrocarbon-degrading bacteria diminishes beyond concentrations of 0.5 to 2.0% (v/v), making it unfavorable for biodegradation processes. As a result, many diesel biodegradation studies focus on concentrations within this range.^30^

The ability of certain bacteria to tolerate high hydrocarbon concentrations is attributed to mechanisms they develop to maintain the integrity of their cell membranes amid a substantial flow of hydrocarbons.^44^ These mechanisms include reducing the content of unsaturated fatty acids in the membrane to increase rigidity, altering the cis/trans conformation of phospholipids, and modifying exclusion systems akin to those used by bacteria for antibiotic resistance.^90^

The structural complexity of the cell wall, particularly in Gram-negative bacteria like *C. violaceum*, is crucial for diesel tolerance. In the work by Babalola et al.,^85^ Gram-negative organisms exhibited greater tolerance to crude oil and diesel toxicity compared to Gram-positive isolates.

Factors such as the pH and temperature significantly influence bacterial growth and play crucial roles in biodegradation processes. Studies^42,79^ have examined the impact of these factors on *C. violaceum* growth, revealing that under certain pH and temperature conditions, this microorganism adjusts the expression of receptors, transporters, and proteins in response to stress. Under stressful conditions, *C. violaceum* demonstrates a higher total protein production but reduced protein diversity, indicating an adaptive response to environmental demands. Temperatures deviating from the species’ optimal growth temperature of 30 °C induce changes in the expression of various proteins, including chaperones and superoxide dismutase, as identified through electrophoresis and mass spectrometry analysis.^42^ Similarly, pH extremes (pH 4 and 9) alter the expression of proteins involved in biosynthetic pathways, molecule recycling, and energy production, impacting the growth rate of *C. violaceum*, which thrives at neutral pH levels.^79^ These findings underscore the intricate molecular responses of *C. violaceum* to environmental stresses, providing insights into its adaptation mechanisms in diverse ecological niches. Variations of the pH and temperature were not conducted in this study. Interaction with other organisms was not studied either. Further research on pH and temperature effects is needed as well as considering immobilization mechanisms and variations in nitrogen sources and interactions with other microorganisms.

## Conclusions

Diesel is a stressful environment for the growth of *C. violaceum* ATCC 12472. Despite this, the strain demonstrates high tolerance and can grow in concentrations as high as 10% (v/v) or 72,500 mg/L, yet diesel causes reductions in specific growth rate and maximum yield of cultures, along with visible changes in colonial morphology and a decrease in the intensity of violacein pigmentation. Among the tested concentrations, the bacteria grew best with 2.5% (v/v) or 18,125 mg/L of diesel. Higher and lower concentrations exhibit inhibitory and stressful effects. From the various kinetic models applied, the Aiba model best represents the use of diesel as a substrate by *C. violaceum*. All inhibition models explain better the relationship between the specific growth rate of *C. violaceum* ATCC 12472 and the initial diesel concentration. This confirms the inhibitory effect of high diesel concentrations on the bacterial growth. According to Luong’s model, the maximum tolerance concentration of *C. violaceum* ATCC 12472 is estimated to be around 12.85% (v/v) or 93,190 mg/L. Finally, the most conducive diesel range for the growth of this bacterium is predicted to be between 2.0 and 3.3% (v/v), equivalent to 14,500 and 23,900 mg/L, respectively.

## Supporting Information Available

Calibration curve of bacterial suspensions, and results of statistical analysis of growth kinetics of *C. violaceum* in diesel (PDF)
