# Supplementary material for: Assessing Diesel Tolerance of Chromobacterium violaceum: Insights from Growth Kinetics, Substrate Utilization, and Implications for Microbial Adaptation
Source: ACS Omega. 2024 May 23;9(22):23741–52. doi: 10.1021/acsomega.4c01698 (PMC11154896; doi:10.1021/acsomega.4c01698)
Supplement: Supplementary file 1 — ao4c01698_si_001.pdf [file ao4c01698_si_001.pdf]

Assessing Diesel Tolerance of *Chromobacterium violaceum*: Insights from Growth Kinetics, Substrate Utilization, and Implications for Microbial Adaptation

Sebastián Arenas [a], Nathaly Rivera [a], Francy Janeth Méndez Casallas [a], Boris Galvis [b,*].

[a] Universidad de La Salle, (https://www.lasalle.edu.co/) Bogotá, Colombia. Programa de Ingeniería ambiental y Sanitaria.

[b] Universidad del Valle, (https://eidenar.univalle.edu.co/) Cali, Colombia. Escuela de Ingeniería de los Recursos Naturales y del Ambiente – EIDENAR.

[*] Email: boris.galvis@correounivalle.edu.co

Supplemental material

STATISTICAL ANALYSES

Growth kinetics of C. violaceum ATCC 12472 at different diesel concentrations

Table S1: Two-factor ANOVA (time and treatment) in the growth kinetics of *C. violaceum* ATCC 12472 in diesel.

Inter-Subject Effects Testing

Dependent variable: Optical Density

Origin	Type III Sum of Squares	gl	Quadratic mean	F	Sig.	Partial Eta Squared
Corrected model	0.940A	83	0.011	398.899	0.000	0.994
Intersection	1.795	1	1.795	63214.485	0.000	0.997
Time	0.440	13	0.034	1192.855	0.000	0.988
Treatment	0.433	5	0.087	3047.313	0.000	0.987
Time * Treatment	0.137	65	0.002	74.256	0.000	0.961
Error	0.006	196	2.839E-5			
Total	2.478	280				
Corrected Total	0.945	279				

[a]. *R squared = 0.994 (Adjusted R squared = 0.992)*

Table S2: Post hoc test for the different treatments evaluated on the growth kinetics of *C. violaceum* ATCC 12472 in diesel.

Optical density

Duncan [a,b,c]

Treatment	N	Subset		
		c	b	to
Control_abiótico	70	0.00601		
7.5 %	42		0.09302	
5 %	42		0.09383	
2.5 %	42			0.09829
1 %	42			0.09874
10 %	42			0.09926
Gis.		1.000	0.472	0.417

The means for the groups are displayed in the homogeneous subsets.
It is based on observed averages.
The error term is the mean square (Error) = 2.839E-5.
[a]. *Use the sample size of the harmonic mean = 45,000.*
[b]. *Group sizes are not equal. The harmonic mean of group sizes is used. Type I error levels are not guaranteed.*
[c]. *Alpha = 0.05.*

Table S3: Post hoc test for the Time factor*Treatment of growth kinetics of *C. violaceum* ATCC 12472 in diesel.

Optical Density

Duncan [a]

Time* Treatment	N	Subset for alpha = 0.05										
		to	b	c	d	and	f	g	h	i	j	k
Day0*2.5%	3	0.009										
Day0*7.5%	3	0.009										
Day0*1%	3	0.010										
Day1*7.5%	3	0.010										
Day1*2.5%	3	0.011										
Day0*10%	3	0.011										
Day0*5%	3	0.012										
Day1*1%	3	0.012										
Day1*5%	3	0.014										
Day1*10%	3	0.014										
Day2*5%	3	0.015										
Day2*10%	3	0.015										
Day2*1%	3	0.016										
Day2*7.5%	3	0.016										
Day2*2.5%	3	0.020										
Day3*10%	3		0.071									
Day3*1%	3		0.078									
Day3*7.5%	3		0.080									
Day3*5%	3			0.090								
Day4*7.5%	3				0.102							
Day4*10%	3				0.102							
Day4*5%	3				0.109							
Day10*2.5%	3				0.112							
Day5*5%	3					0.123						
Day5*7.5%	3					0.123						
Day4*1%	3					0.125						
Day5*10%	3					0.126						
Day3*2.5%	3					0.128						
Day10*7.5%	3					0.128						
Day10*5%	3					0.129						
Day10*1%	3					0.130						
Day9*2.5%	3						0.139					
Day4*2.5%	3						0.140					
Day7*5%	3						0.141	0.141				
Day10*10%	3						0.141	0.141				
Day5*1%	3						0.142	0.142				
Day9*5%	3						0.143	0.143	0.143			
Day9*7.5%	3						0.144	0.144	0.144	0.144		
Day8*5%	3						0.147	0.147	0.147	0.147	0.147	
Day8*7.5%	3						0.147	0.147	0.147	0.147	0.147	
Day5*2.5%	3						0.147	0.147	0.147	0.147	0.147	
Day7*7.5%	3						0.149	0.149	0.149	0.149	0.149	0.149
Day9*1%	3						0.149	0.149	0.149	0.149	0.149	0.149
Day8*2.5%	3						0.151	0.151	0.151	0.151	0.151	0.151
Day7*1%	3							0.153	0.153	0.153	0.153	0.153
Day7*10%	3								0.154	0.154	0.154	0.154
Day7*2.5%	3									0.155	0.155	0.155
Day8*1%	3										0.157	0.157
Day8*10%	3										0.158	0.158
Day9*10%	3											0.160
Gis.		0.065	0.070	1.000	0.070	0.225	0.055	0.053	0.050	0.066	0.068	0.055

The means for the groups are displayed in the homogeneous subsets.

[a]. *Use the sample size of the harmonic mean = 3000*

Growth kinetics of C. violaceum ATCC 12472 in LB broth

Table S4: ANOVA of the optical density over time of the growth kinetics of *C. violaceum* ATCC 12472 in LB broth.

ANOVA

Optical Density

	Sum of squares	gl	Quadratic mean	F	sig.
Between groups	2.526	24	0.105	139.557	0.000
Within Groups	0.038	50	0.001		
Total	2.564	74			

Table S5: Post hoc test for the optical density over time of the growth kinetics of *C. violaceum* ATCC 12472 in LB broth.

Optical Density

Duncan [a]

Time	N	Subset for alpha = 0.05							
		to	b	c	d	and	f	g	h
0	3	0.03644							
1	3	0.05447							
2	3	0.07416	0.07416						
3	3		0.11582						
4	3			0.29835					
5	3				0.35879				
6	3					0.45156			
7	3					0.48087	0.48087		
8	3						0.50309	0.50309	
9	3							0.54011	0.54011
10	3							0.54978	0.54978
11	3								0.55980
12	3								0.56277
22	3								0.57078
15	3								0.57159
13	3								0.57199
16	3								0.57204
18	3								0.57284
24	3								0.57365
14	3								0.57577
20	3								0.57577
23	3								0.57577
19	3								0.57662
17	3								0.57788
21	3								0.57828
Gis.		0.118	0.069	1.000	1.000	0.197	0.327	0.053	0.168

The means for the groups are displayed in the homogeneous subsets.
[a]. *Use the sample size of the harmonic mean = 3000.*

Kinetic Growth Parameters of C. violaceum ATCC 12472 in Diesel and LB Broth

Table S6: ANOVA of the kinetic growth parameters of *C. violaceum* ATCC 12472 in diesel and LB broth.

ANOVA						
		Sum of squares	Gl	Quadratic mean	F	Gis.
Vmax	Between groups	1.291	5	0.258	149.036	0.000
	Within Groups	0.021	12	0.002		
	Total	1.311	17			
Tg	Between groups	373.046	5	74.609	18.491	0.000
	Within Groups	48.418	12	4.035		
	Total	421.464	17			
M	Between groups	0.373	5	0.075	2494.351	0.000
	Within Groups	0.000	12	0.000		
	Total	0.373	17			

Table S7: Post hoc test for the kinetic growth parameters of *C. violaceum* ATCC 12472 in diesel and LB broth.

Max Vel

Duncan [a]

Treatment	N	Subset for alpha = 0.05		
		to	b	c
10 %	3	0.0490		
7.5 %	3	0.0575		
1 %	3	0.0889	0.0889	
5 %	3	0.0968	0.0968	
2.5 %	3		0.1546	
LB Broth	3			0.8020
Gis.		0.216	0.090	1.000

Tg

Duncan [a]

Treatment	N	Subset for alpha = 0.05		
		c	b	to
LB Broth	3	0.8716		
2.5 %	3		4.5480	
5 %	3		7.7732	
1 %	3		7.9656	
7.5 %	3			12.5942
10 %	3			14.3609
Gis.		1.000	0.070	0.303

M

Duncan [a]

Treatment	N	Subset for alpha = 0.05			
		to	b	c	d
5 %	3	0.12363			
7.5 %	3		0.13527		
1 %	3		0.13872	0.13872	
2.5 %	3		0.14305	0.14305	
10 %	3			0.14796	
LB Broth	3				0.52327
Gis.		1.000	0.123	0.071	1.000

The means for the groups are displayed in the homogeneous subsets.

[a]. Use the sample size of the harmonic mean = 3000.
